# Open-label placebos reduce hair cortisol concentrations and psychological distress - a randomized controlled trial

**DOI:** 10.1038/s41598-026-54791-8

**Published:** 2026-06-14

**Authors:** Carolin Liedtke, Michael Schaefer, Sören Enge

**Affiliations:** 1 Department of Psychology, MSB Medical School Berlin, Rüdesheimer Straße 50, 14197 Berlin, Germany; 2 Institute of Neuroscience and Biopsychology for Clinical Application, MSB Medical School Berlin, Berlin, Germany

**Keywords:** Open-label placebo, Hair cortisol, Psychological distress, Negative affect, Randomized controlled trial, Biomarkers, Health care, Psychology, Psychology

## Abstract

The exposure to stressful events, such as demanding university exams, is associated with psychological distress and elevated physiological stress responses, which may impair performance. Recent research suggests that open-label placebo (OLP) treatments can effectively reduce psychological distress. However, evidence regarding OLP effects on biomarkers remains limited, highlighting the need for systematic investigation. This study presents the first longitudinal randomized controlled trial examining a four-week OLP intervention on hair cortisol concentrations (HCC) and psychological distress, using oral university exams as real-life stressors. 134 healthy university students were randomized to an OLP group receiving placebo pills or a control group without treatment. Psychological distress was assessed via a latent factor derived from area under the curve (AUCi) values of negative affect, test anxiety, subjective stress, and exam-related stress, continuously measured during the intervention. Analyses revealed decreasing HCC in the OLP group, demonstrating for the first time a long-term physiological effect of OLPs comparable to other stress-management interventions. Results further indicate reduced psychological distress in the OLP group. Pre-exam negative affect mediated subsequent improvements in exam grades observed in the OLP group, compared to the controls. These findings provide first evidence that OLPs may attenuate physiological and psychological impacts of prolonged stress exposure.

Trial registration: This study was registered at the German clinical trials register (DRKS00031423).

## Introduction

The placebo effect can be defined as a psychological or psychophysiological change caused by inactive or nonspecific treatments, such as inert pills or substances without specific effects on the condition being treated^1^. In medical research, placebos typically serve as control conditions to evaluate the efficacy of drugs or interventions. Beyond this, placebos demonstrate potential therapeutic benefits^2^, such as alleviating stress, anxiety symptoms^3^, or pain^4^, which may explain their common prescription in routine clinical practice^5^. However, the usual deceptive administration of placebos, where patients believe that they receive an active treatment, is critically discussed with respect to informed consent and patient autonomy^6^.

Remarkably, recent findings suggest that even placebos without deception, so-called open-label placebo (OLP) treatments, in which individuals are transparently informed that they receive a placebo, can yield beneficial effects in various conditions, including irritable bowel syndrome^7^, allergic symptoms^8^, and chronic back pain^9^. Moreover, there is evidence that OLPs may be similarly effective to deceptive placebos^7^, and meta-analyses revealed small to medium-sized effects of OLPs on diverse symptoms in clinical and non-clinical samples^10–12^, supporting their potential therapeutic benefits.

In recent years, a significant increase in psychological distress^13^ and a generally high prevalence of mental health problems^14^ has been observed among university students. Psychological distress manifests in various affective states, such as stress, anxiety, anger, or depressed mood, and is commonly conceptualized as a latent construct within multivariate psychometric frameworks. Situational influences, such as failure to achieve personal goals or good performances—both of which may be prevalent in university settings—as well as cognitive appraisals of inadequate coping or a lack of personal control over significant events can contribute to heightened levels of psychological distress^15^. Furthermore, increasing academic demands may contribute to chronic stress^16^ and, particularly during exam periods, heighten negative affect^17^. As a consequence, psychological distress may negatively impact academic performance^18^.

In this context, placebos may be a promising treatment approach, as studies on conventional placebos have demonstrated beneficial effects on stress, anxiety, and depressive symptoms^3,19,20^. Moreover, OLP treatments have also been shown to reduce symptoms of psychological distress in non-clinical populations^21–27^. El Brihi et al.^21^ reported significantly lower distress in the intervention group after OLP administration compared with a no-treatment control. Evidence also suggests that OLPs may reduce depressive symptoms^22^, perceived stress^23^, and anxiety^24,25^. In academic settings, OLPs have been linked to reduced test anxiety^26,27^, which affects up to 52% of university students^28,29^, highlighting OLPs as an important treatment possibility.

However, there is limited evidence that OLP treatments lead to improved objective parameters, such as physiological distress measures or performance indicators^23–27,30^. While Guevarra et al.^30^ demonstrated that non-deceptive placebos modulate electroencephalographic measures of emotional distress, Schaefer et al.^24^ reported effects of OLPs on salivary cortisol levels only among participants with strong placebo beliefs. Other studies found no significant effects of OLPs on physiological outcomes^31–33^. Overall, a recent meta-analysis found no consistent positive or negative effect of OLP effects on objective measures, highlighting a substantial need for further research, particularly given the scarcity of studies investigating physiological or other objective outcomes^12^.

In stress research, hair cortisol concentration (HCC) is an increasingly used biomarker for assessing retrospective, long-term cortisol levels^34^. Cortisol incorporated into hair provides a non-invasive measure of the hypothalamic-pituitary-adrenocortical (HPA) axis activity over extended periods^35^. With 22 to 43% elevated cortisol levels under present and ongoing stress exposure^36^, HCC proved to be a sensitive parameter to capture longitudinal cortisol secretion. Supporting its validity as a biomarker of chronic or cumulative stress, HCC has been associated with long-term psychological strain^37^, perceived stress^38^, serious adverse life events, trauma exposition, and stress-related psychiatric disorders^36^. HCC increases during the academic term^39^, emphasizing the psychophysiological impact of academic stress and supporting HCC as a valid indicator of cumulative stress.

The impact of OLPs on long-term cortisol levels has yet to be investigated. Existing studies with small samples that focused on acute salivary cortisol levels in laboratory stress tests or pain paradigms have yielded inconsistent results^24,31,33^. Given that stressors involving performance demands and social-evaluative threat elicit stronger HPA axis responses than standard laboratory tasks^40^, an oral university exam represents a strong and effective naturalistic stressor^41^. While a few OLP studies have been conducted in exam-related settings^23,26,27^, they did not explicitly investigate oral exams (e.g., used written midterm exams) and did not include any physiological measures.

Physiological measures provide essential, objective insights into emotional reactivity that self-reports alone cannot fully capture^42^. As exposure to stressful events affects multiple physiological systems (e.g., autonomic nervous system, HPA axis, immune system^43^), quantifying physiological stress responses is urgently needed to understand the multi-level effects of OLP treatments. Considering that deceptive placebos can modulate physiological indicators^44,45^, it is of importance to examine whether and to what extent OLPs affect psychophysiological stress responses in a real-life stress scenario.

The reasons behind the effects of OLP treatments remain largely unclear. Some researchers argue that associative learning and classical conditioning may represent possible underlying mechanisms^46^, while others emphasize the role of positive treatment expectation^47^. Several studies have investigated treatment expectations or the belief in (open-label) placebos in relation to the OLP effect, but results have been mixed^24,25,30,48^. Compared to deceptive placebos, the role of expectation and belief in OLPs is less clear, since participants are explicitly informed that they are receiving inert pills as treatment. This issue requires further investigation.

The aim of the present study was to investigate the OLP effects on HCC and measures of psychological distress in a randomized controlled trial (RCT) using an oral university exam as a real-life stressor. We expect the OLP intervention to lead to lower HCC and psychological distress during the intervention period, compared to no-treatment controls. Given the multifaceted nature of psychological distress, we selected domain-relevant indicators for its assessment. In the present study, psychological distress is therefore operationalized as a latent construct reflected by negative affect, test anxiety, subjective stress, and exam-related stress. By capturing the shared variance across the multiple instruments, the latent construct reflects the underlying distress experience while improving construct validity and reliability, leading to increased statistical power in subsequent statistical analyses^49,50^. By continuously assessing measures of psychological distress during the one-month intervention, this study also allows us to consider the temporal dynamics of the OLP effect. Psychological distress may increase in all participants until the exam but is expected to be lower in the OLP group. We also investigate whether the potential OLP effect persists until the exam day, considering the affective states immediately before the exam. In this context, it is also of interest whether group differences in negative affect may indirectly influence exam performance, thereby supporting a mediation pathway. Furthermore, the role of placebo belief and expectations will be investigated.

## Methods

### Sample

**Fig. 1:**
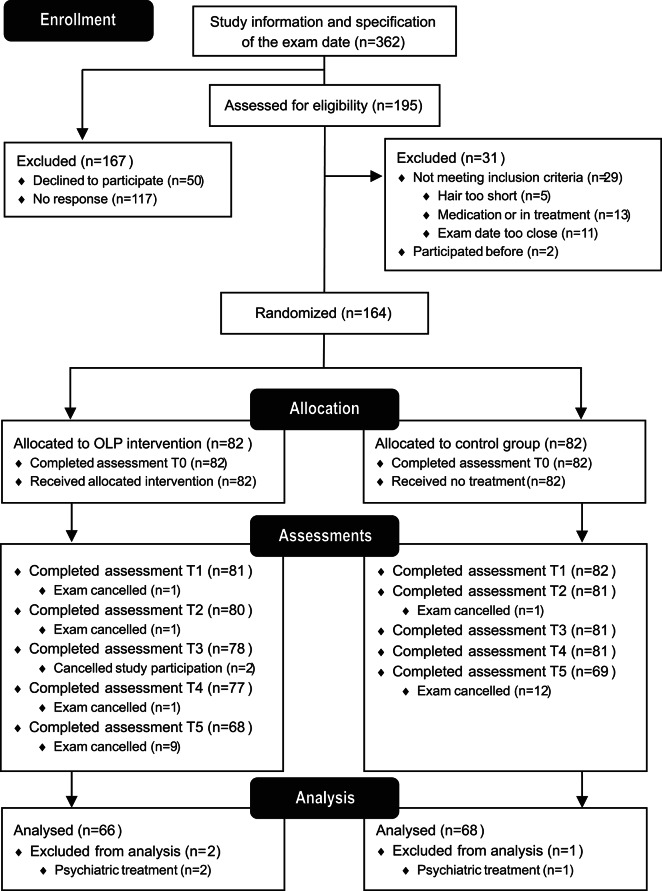
CONSORT flowchart of participants.

A total of 134 healthy German university students completed the trial (mean age 26.87 ± 4.67 years, 104 females [77.6%], OLP group = 66 [49.3%], control group = 68 [50.7%]; see Fig. 1 for CONSORT flowchart). Inclusion criteria were the completion of an oral university exam at the end of the term and a minimum hair length of 3 cm. Exclusion criteria were current psychiatric, neurological, or endocrinological diseases and treatments, diabetes, the use of corticosteroid medication, allergies to sugar, artificial additives, or colorings, heavy smoking (> 10 cigarettes/day), regular consumption of illegal drugs, and pregnancy. Three participants who reported undergoing psychiatric treatment were excluded from the analyses. After completing the trial, the control group had the opportunity to obtain placebo pills, and all participants received a remuneration of 40 euros and 6 h of course credits.

### Experimental design

In this RCT, healthy students were assigned to either the OLP intervention or a no-treatment control group. The study was approved by the ethical board of the MSB Medical School Berlin (dossier MSB-2021/60) and conducted in accordance with the Declaration of Helsinki. Prior to the study, each participant provided written informed consent and was informed about the voluntary nature of the study participation, the possibility to withdraw from participation at any time, and the pseudonymized processing of their personal data. This project is funded by the Deutsche Forschungsgemeinschaft (DFG, German Research Foundation, Project-ID 505631897, EN 1155/3 − 1) and registered at the German clinical trials register (DRKS00031423, registered on 07/03/2023). Based on effect sizes reported in previous meta-analyses^10–12^, we calculated an inverse-variance-weighted average (as described in^51^), resulting in an estimated effect size of *f* = 0.28. Based on this estimate, an a priori power analysis using G*Power indicated a required total sample size of *N* = 103, assuming an α error probability of 0.05 and a desired power of 0.8.

### Study procedure

**Fig. 2:**
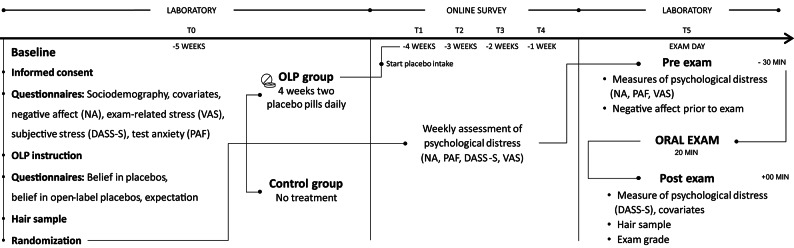
Study procedure timeline.

Five weeks before the exam (T0), sociodemographic characteristics, potential covariates, and baseline values of psychological distress measures were assessed via questionnaires (see Fig. 2 for study procedure timeline). Adopted from previous OLP studies^22,25,26,52^, all participants were provided with a standardized written instruction describing the OLP rationale. This instruction included the following statements: (1) A placebo is an inactive substance without medical ingredients, but they may still be effective, as shown in various studies; (2) a possible mechanism may be classical conditioning; (3) for the placebo effect, a positive attitude may be helpful but not necessary; (4) it is important to take the placebo pills faithfully. The participants were further informed that OLPs may have positive effects and watched a television news report explaining OLPs (analogous to^9,25^). Subsequently, participants reported their belief in (open-label) placebos and their treatment expectation and provided the first hair sample. After these assessments, randomization was conducted via sealed opaque envelopes, assigning participants in a 1:1 ratio to either the OLP or control group. Assistants of the principal investigators conducted the randomization. Due to the non-deceptive placebo intervention, the participants were informed about their group assignment. The researchers and principal investigators were blinded regarding the group assignment.

The OLP group received a package with 56 placebo pills (“P-pills”, white, 7 mm, Lichtenstein, manufactured by Zentiva Pharma GmbH), labeled with the university logo and the instruction to take two pills daily for four weeks (one in the morning and one in the evening). Participants in the OLP group were instructed to take the first placebo pill 28 days before the exam in the evening and the last pill on exam day in the morning. To maintain comparable participant-experimenter interaction and contact duration across groups, participants in the OLP group were regularly reminded to take their placebo pills as part of the weekly email questionnaires sent to all participants. The control group participants received no treatment but were informed about the importance of the control condition in RCTs and were offered the opportunity to receive placebo pills after completing the trial.

In the following four weeks, measures of psychological distress were assessed continuously (28 [T1], 21 [T2], 14 [T3], and 7 days [T4] prior to the exam) for both groups via online questionnaires. On exam day (T5), participants completed questionnaires about their affective states 30 min before and immediately after the 20-minute oral exam, reported their exam grade, and provided the second hair sample. Participants in the control group were asked about potential feelings of disappointment with respect to their group assignment. To measure adherence, the OLP group was asked to return their placebo package with leftover pills. In accordance with clinical trials, ≥ 80% can be defined as good adherence^53^.

### Outcome measures

#### Primary outcomes

To collect the hair samples, two thin hair strands from the posterior vertex were isolated with a loop, then cut closely to the scalp (analogous to^34,54^) and stored in an aluminum envelope. We took the hair samples at two times, 35 days before the exam [T0] and on exam day [T5]. The HCC was determined from the 1 cm long segment closest to the scalp. Given an average human hair growth of approximately 1 cm per month^55^, the T5 segment reflects the HCC during the intervention period, while the T0 segment represents the pre-intervention HCC and serves as a reference value. HCC was determined by liquid chromatography coupled with tandem mass spectrometry (LC-MS/MS), a highly selective analytical technique to, e.g., detect various corticosteroids in hair^56^. The hair sample preparation and cortisol extraction were conducted by an independent laboratory (Dresden LabService GmbH, Dresden, Germany), following the protocol described in Gao et al.^34^. The limit of quantification (LOQ) was below 0.3 picogram/milligram (pg/mg), and the intra-assay coefficients of variance were between 3.8 and 12%.

Psychological distress was operationalized using different self-report measures, assessing negative affect, test anxiety, subjective stress, and exam-related stress. Negative affect was measured using the German version of the Positive and Negative Affect Schedule (PANAS)^57^, specifically the 10-item negative affect (NA) subscale, which assesses emotions like dejection, anger, and anxiety, related to the past week during the intervention month and to the moment immediately before the exam. Test anxiety was measured using the 20-item German Text Anxiety Inventory (PAF)^58^. The subscales worry, emotionality, interference, and lack of confidence refer to possible feelings and thoughts related to exam situations. The 7-item subscale stress of the German Depression Anxiety and Stress Scale 21 (DASS-21)^59^ was used to assess subjective stress (DASS-S) related to the past week. Additionally, a visual analogue scale (VAS, range 0-100)^60^ was used to assess exam-related stress, i.e., how stressful the participants perceive the current situation with regard to the exam.

#### Secondary outcomes

The exam grades (range 1–5) were documented as an objective performance measure, with 1 = “very good”, 4 = “sufficient”, and 5 = “failed”. The general belief in placebos (as described in^48^) and the belief in OLPs (as described in^30^) were assessed prior to randomization using statements drawn from these respective studies. Moreover, the participants indicated on a VAS scale (range 0-100) to what extent they expected the placebo pills to reduce their tension or anxiety during the exam.

### Statistical analysis

#### Data preprocessing and latent variable modeling

The HCC values were log-transformed due to positively skewed raw data (e.g.,^54,61^). Then we computed a change score that indicates the change in HCC from baseline to the intervention month, with negative values reflecting a decrease and positive values an increase in cortisol levels. For each self-report measure (NA, PAF, DASS-S, VAS), the area under the curve with respect to increase (AUCi) was calculated, a widely used approach in stress research (e.g.,^62^) that provides a summarizing indicator of changes over time while preserving information from repeated measurements; thus, the AUCi quantifies fluctuations during the intervention (T1 to T5). By incorporating both intensity and sensitivity of reactivity, the AUCi is particularly useful for examining responses to specific challenges and stressors^63,64^. As higher baseline values are typically associated with lower AUCi scores^63^, which may distort the interpretation of reactivity, we residualized the AUCi values by regressing them on their respective baseline values to control for potential baseline differences. The resulting standardized residuals, which are independent of baseline variance, were used in all subsequent analyses.

Next, a confirmatory factor analysis (CFA) was conducted to model a latent variable representing psychological distress over the four-week period of OLP treatment. A single-factor model was specified, in which the four AUCi values (NA, PAF, DASS-S, and VAS AUCi) were hypothesized to load on this factor, with negative affect (NA AUCi) serving as a marker variable. No missing values and multivariate outliers were present in the dataset. Due to violations of multivariate normality, a robust maximum likelihood (MLR) method was applied for parameter estimation. The model fit was evaluated based on commonly used fit indices, including the comparative fit index (CFI), the Tucker-Lewis index (TLI), the root mean square error of approximation (RMSEA), the standardized root mean square residuals (SRMR), and the χ² statistic. Conventional cutoff values (CFI ≥ 0.95, TLI ≥ 0.95, RMSEA ≤ 0.06, SRMR ≤ 0.08) were used to determine model adequacy^65^. The CFA was conducted using the lavaan package in R (version 4.3.2).

#### Data analysis

Demographic characteristics and baseline values before starting the intervention (T0) were tested for group differences. To examine the effects of the OLP intervention, separate analyses of covariance (ANCOVAs) were conducted, with group (OLP vs. control) as between-subject factor and the primary outcomes as dependent variables. The primary outcomes were the latent AUCi factor representing psychological distress during the intervention month, the HCC change score, and negative affect (NA) on the exam day, assessed prior to the exam (-30 min). All analyses included age and sex as covariates given their potential influence on psychological distress, negative affect, and hair cortisol^66–68^. Regarding the model with HCC as outcome measure, possible confounders (including body mass index [BMI], frequency of hair washes, ultraviolet exposition, physical activity, hair weight, hormonal contraceptives, and job hours) and the HCC baseline value were included as additional covariates based on previous findings^36,54,61,68–70^. In line with previous studies on steroid hormones, HCC cutoff levels in baseline were defined as the 2.5th and 97.5th percentiles^71–74^. Due to missing cases in HCC (*n* = 1) and relevant covariates (*n* = 5), as well as the exclusion of outliers beyond the cutoff levels (*n* = 4), this analysis includes a sample of *n* = 124. The descriptive information is reported in the original units (pg/mg).

To test whether OLP effects may indirectly influence exam performance by reducing negative affect before the exam, a mediation analysis was performed. The bootstrapping method, including 5000 resamples, was used to calculate 95% confidence intervals (CI) for the indirect effect. Due to the dichotomous independent variable (0 = OLP, 1 = control), partially standardized effects are reported for this analysis. In the OLP group, partial correlation analyses (Spearman’s rho) were conducted to examine whether the participants’ belief in (open-label) placebos and their expectations about the treatment were associated with potential OLP effects in the primary outcomes. Finally, exploratory point-biserial correlation analyses were conducted in the control group to examine whether reported disappointment about group allocation was associated with the primary outcome measures. The analyses were performed with SPSS (version 27.0), applying two-tailed tests with a significance level of *α* = 0.05.

## Results

**Fig. 3:**
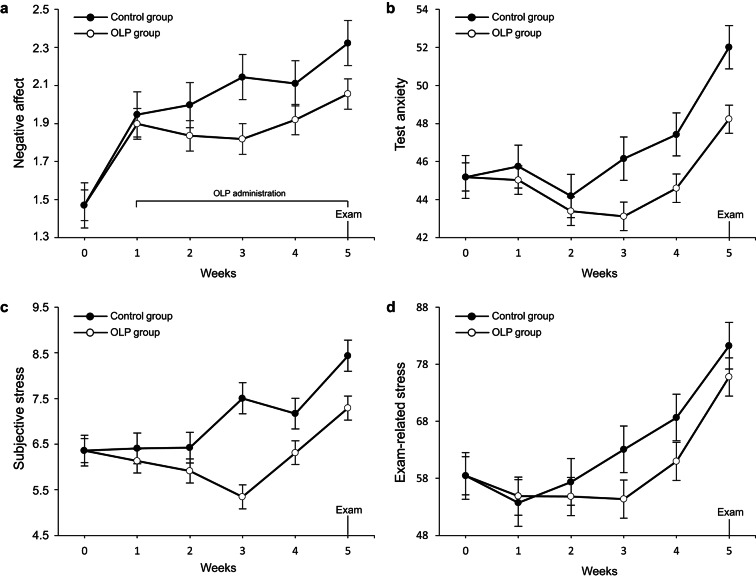
Psychological distress measures from baseline to exam day. (**a**) Negative affect (NA), (**b**) test anxiety (PAF), (**c**) subjective stress (DASS-S) and (**d**) exam-related stress (VAS) over five weeks until an oral exam. Line plots represent adjusted means of OLP (*n* = 66) and control group (*n* = 68) controlled for sex, age and baseline differences. Error bars represent ± 1 standard error of the mean.

Demographic and baseline characteristics are presented in Table 1. Before starting the intervention, the OLP (*n* = 66) and control group (*n* = 68) did not differ significantly in the baseline values and demographic characteristics (all *p* > .05), except for the VAS baseline (*p* = .030) and sex (*p* = .048). However, these differences were statistically controlled for by baseline-corrected AUCi values and by considering sex as a covariate in the main analyses. The overall adherence in the OLP group was 97.0 ± 5.49%, indicating that participants adhered well to the OLP regimen. The development of psychological distress measures during the intervention month is shown in Fig. 3.

**Table 1:** Demographic and baseline characteristics for OLP and control group.

Characteristic	OLP group		Control group		*p*	
*n*	66		68			
Age (in years)	26.09 ± 4.18		27.62 ± 5.02		*p* = .058	
Females/males^1^	56/10		48/20		*p* = .048	
Study semester^2^	1.86 ± 1.04		1.91 ± 0.99		*p* = .667	
Grade average^2^	1.81 ± 0.39		1.91 ± 0.51		*p* = .355	
Belief in placebos	6.75 ± 1.81		6.80 ± 1.95		*p* = .884	
Belief in OLPs	6.21 ± 2.30		6.94 ± 2.25		*p* = .063	
Expectation	28.33 ± 15.98		31.68 ± 20.21		*p* = .289	
HCC (T0)	4.10 ± 2.67		5.53 ± 5.01		*p* = .067	

	T0	T1	T0	T1	T0	T1
Negative affect (NA)^a^	1.45 ± 0.45	1.90 ± 0.59	1.49 ± 0.61	1.95 ± 0.73	*p* = .679	*p* = .653
Test anxiety (PAF)^b^	44.05 ± 10.98	44.36 ± 9.69	46.29 ± 12.40	46.38 ± 10.49	*p* = .269	*p* = .249
Subjective stress (DASS-S)^c^	5.73 ± 3.87	5.70 ± 3.62	6.97 ± 4.42	6.82 ± 4.29	*p* = .086	*p* = .103
Exam-related stress (VAS)^d^	52.65 ± 31.30	52.17 ± 27.27	64.04 ± 28.82	56.34 ± 27.89	*p* = .030	*p* = .383

Mean ± 1 standard deviation is reported. Results for t-test, ^1^χ^2^ test, and ^2^Mann-Whitney-U-test are shown in *p* values. T0 = assessment before randomization; T1 = assessment one week after randomization, first day of OLP intake.

^a^Assessed related to the moment (T0) and to the past week (T1). ^b^Assessed in general (T0) and with regard to the upcoming exam (T1). ^c^Assessed related to the past week. ^d^Assessed for the current situation with regard to the exam.

**Fig. 4:**
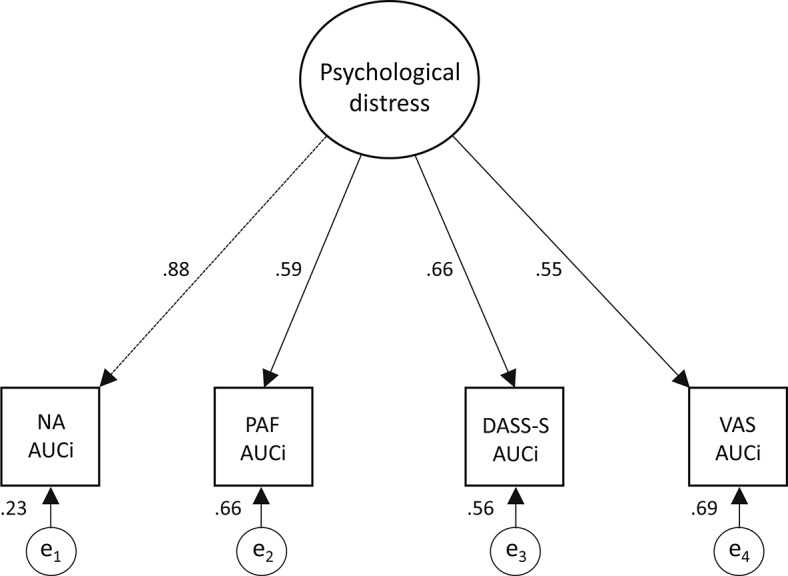
Confirmatory factor analysis (CFA) of psychological distress. All standardized estimates are significant (*p* < .01). Indicator variables are the area under the curve (AUCi) values of negative affect (NA), test anxiety (PAF), subjective stress (DASS-S) and exam-related stress (VAS).

Correlation analyses conducted prior to the CFA revealed statistically significant moderate to large associations between the AUCi values of self-report measures (NA, PAF, DASS-S, VAS), ranging from *r* = 0.330 to 0.578 (Spearman’s rho, all *p* < .001). The CFA based on these AUCi values (see Fig. 4) demonstrated an excellent model fit (χ²(2) = 0.781, *p* = .677; robust CFI = 1.000; robust TLI = 1.032; robust RMSEA = 0.000 [90% CI: 0.000, 0.139]; SRMR = 0.015). All standardized factor loadings ranged from 0.55 to 0.88 (*p* < .001), supporting adequate indicator reliability. The internal consistency of the latent factor was acceptable (composite reliability = 0.77, McDonald’s ω = 0.77). The factor was subsequently used to examine OLP effects on psychological distress.

### OLP effect during the intervention month

**Fig. 5:**
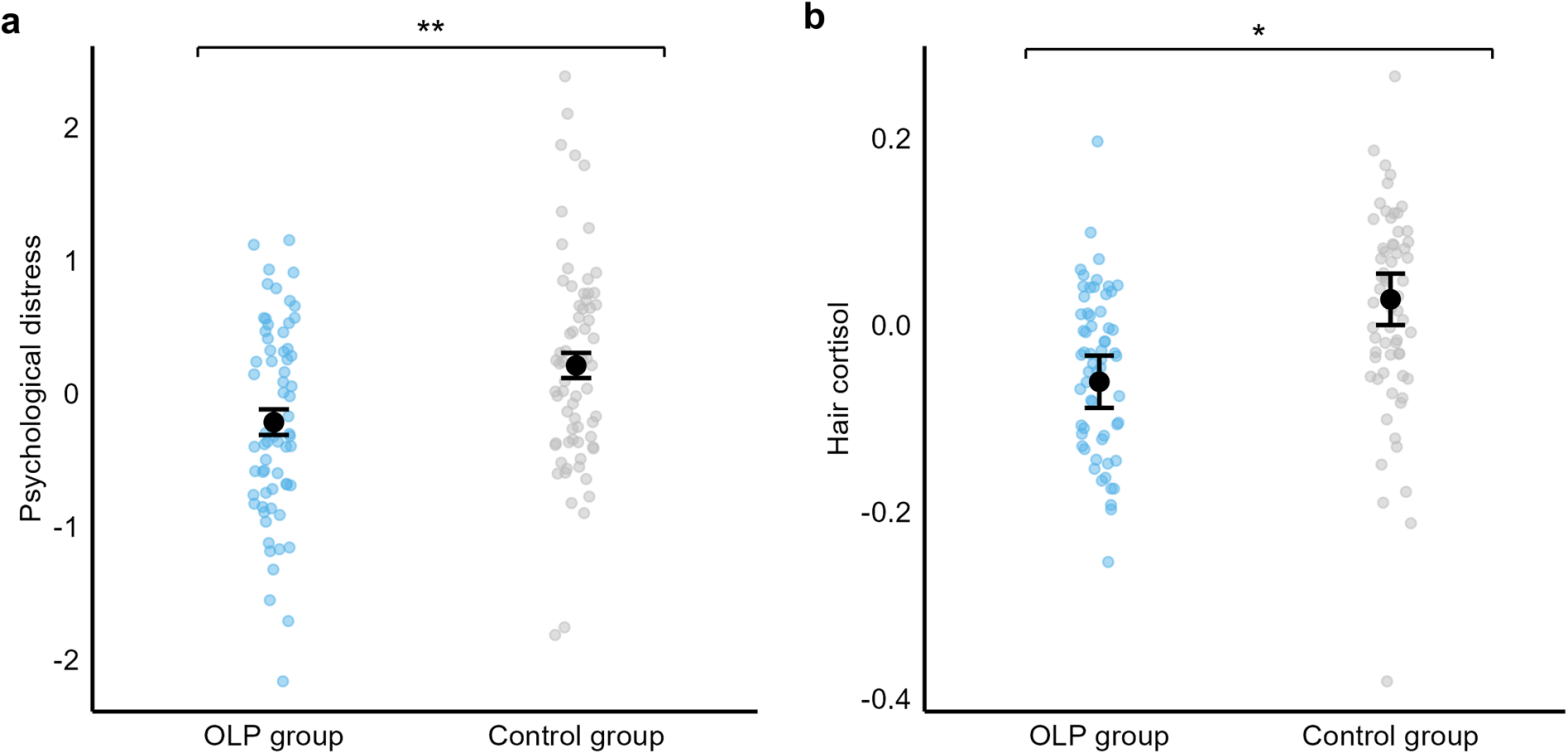
Changes in (**a**) psychological distress and (**b**) logarithmized HCC during the OLP intervention month. Plots represent estimated marginal means ± 1 standard error of the mean for OLP and control group derived from ANCOVAs. Jittered points (blue and grey) illustrate individual data points per group. *p < .05, ** *p* < .01.

Results of the ANCOVA revealed significant group differences with a medium effect size in psychological distress during the four-week intervention period (*F*[1, 130] = 9.722, *p* = .002, $\:{\eta\:}_{p}^{2}$ = 0.070; Fig. 5a). Adjusted means showed lower psychological distress in the OLP group (*M*_*adjusted*_ = − 0.216, *SE* = 0.096), compared to the control group (*M*_*adjusted*_ = 0.210, *SE* = 0.095).

During the intervention interval, HCC in the OLP group ranged from 0.39 to 16.00 pg/mg (*M* = 3.79, *SD* = 2.82), while in the control group HCC ranged from 1.09 to 53.11 pg/mg (*M* = 5.97, *SD* = 7.01). The ANCOVA (OLP [*n* = 61], control [*n* = 63]) revealed significant group differences with a small-to-medium effect size (*F*[1, 111] = 4.823, *p* = .030, $\:{\eta\:}_{p}^{2}$ = 0.042). As depicted in Fig. 5b, the OLP group showed negative change scores indicating decreasing HCC values during the intervention compared to the pre-intervention interval (*M*_*adjusted*_ = − 0.061, *SE* = 0.028), while increasing HCC was observed for the control group (*M*_*adjusted*_ = 0.027, *SE* = 0.027).

### OLP effect on exam day

**Fig. 6:**
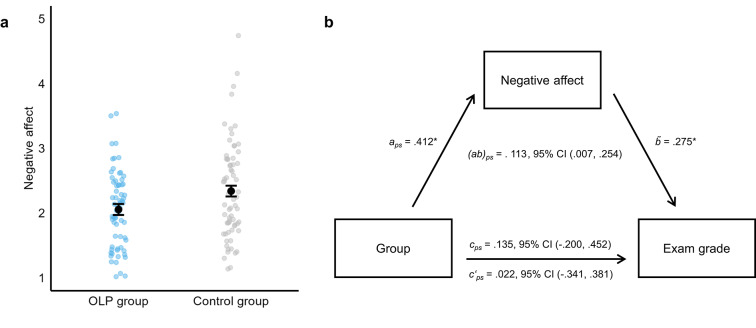
OLP effect on exam day. (**a**) Estimated marginal means ± 1 standard error of the mean of negative affect immediately before the oral exam for OLP and control group derived from ANCOVA. Jittered points (blue and grey) illustrate individual data points per group. (**b**) Relationship between group (0 = OLP, 1 = control) and the exam grade mediated by pre-exam negative affect. Standardized (b-path), partially standardized effects (a, ab, c, c’-paths) and percentile confidence intervals (CI) are reported. *p < .05.

Negative affect state (NA) measured immediately before the exam (-30 min) revealed a significant, small-to-medium-sized difference between the groups in an ANCOVA (*F*[1, 130] = 5.535, *p* = .020, $\:{\eta\:}_{p}^{2}$ = 0.041; Fig. 6a), showing lower negative affect in the OLP group (*M*_*adjusted*_ = 2.055, *SE* = 0.085) compared to the control group (*M*_*adjusted*_ = 2.339, *SE* = 0.084). Moreover, the pre-exam negative affect was significantly correlated with the exam grades (*r* = .277, *p* = .001). The mediation model (Fig. 6b) revealed a positive indirect effect of group on the exam grades through negative affect (*ab*_*ps*_ = 0.113, 95% percentile CI [0.007, 0.254]), indicating better exam grades in the OLP group mediated by a lower negative affect state. The total effect of the group on exam grades (*c*_*ps*_ = 0.135, 95% percentile CI [-0.200, 0.452]), as well as the direct effect controlled for negative affect (*c’*_*ps*_ = 0.022, 95% percentile CI [-0.341, 0.381]), were not significant. Thus, the effect of group on exam grade is mediated through the indirect pathway of negative affect.

### Belief and expectation

In the OLP group, no significant correlations were found between the primary outcome measures (change in HCC and psychological distress, pre-exam negative affect) and the variables general belief in placebos (*r* = − 0.121 to − 0.140, all *p* > .30), belief in OLPs (*r* = 0.016 to − 0.055, all *p* > .80), or treatment expectations (*r* = 0.201 to − 0.059, all *p* > .10). General belief in placebos and belief in OLPs were not significantly associated in the total sample (*r* = 0.038, *p* = .661). In the control group, no significant correlations were found between the primary outcome measures and disappointment (*r* = − 0.052 to 0.086, all *p* > .50).

## Discussion

This study examined the effects of OLPs on self-reported psychological distress and LC-MS/MS-based hair cortisol concentrations as a physiological long-term measure, using a real-life stressor and a longitudinal perspective. Results revealed that a one-month OLP intervention before an oral university exam reduced psychological distress, indicating the effectiveness of OLPs in attenuating the impact of prolonged stress exposure. This aligns with prior research demonstrating that OLPs diminish self-assessment of stress^23^, test anxiety^25–27^, and depressive symptoms^22^, and with evidence that conventional deceptive placebos alleviate psychological distress^3^. By modeling psychological distress as a latent construct based on AUCi values of four related indicators (negative affect, test anxiety, subjective stress, and exam-related stress), we controlled measurement errors and obtained a more robust estimate of the participants’ distress experience^75^. Moreover, the group effect (OLP vs. control) revealed a medium effect size, comparable to those found in other OLP studies with healthy subjects^12^, as well as in web-based interventions (e.g., cognitive behavioral therapy programs) targeting stress and anxiety among university students^76^. Taking into account that highly stressed students more frequently use medication to enhance mood and cognitive performance^77^, which may entail adverse side effects and health risks^78^, our findings provide meaningful evidence that OLPs are an effective and safe treatment option for distressed students^13^ and for stress-related symptoms in general.

Intriguingly, this study is the first to demonstrate an OLP effect on a physiological long-term stress measure. The OLP treatment significantly reduced HCC during the intervention month compared to the control group, with a small-to-medium effect size. This suggests that OLPs appear to be similarly effective in reducing long-term stress—measured by HCC—as other psychological interventions, such as mindfulness-based or stress reduction programs for healthy individuals^79,80^. In contrast to salivary cortisol levels, which were used in previous OLP studies^24,31,33^, HCC represents a more stable and reliable indicator of the long-term HPA axis activity, being less susceptible to situational and diurnal variations^35^. Given that the HPA axis is particularly responsive to social-evaluative threat^40^ and that HCC is associated with stressful events involving academic demands^39^, it is noteworthy that the present OLP treatment led to a measurable reduction in long-term cortisol levels during the course of university exams. This indicates that the benefits of OLPs may extend beyond subjective evaluations, highlighting their potential to improve both psychological and physiological stress-related outcomes.

The OLP effects were sustained until the exam, as evidenced by significantly lower negative affect in the OLP group immediately prior to the exam, with a small-to-medium effect size. Since previous OLP studies that used university exams as real-life stressors did not explicitly investigate the emotional states on the exam day^23,26,27^, our findings provide relevant new insights into the effectiveness and maintenance of the OLP effect under acute academic stress.

Furthermore, state negative affect assessed directly before the exam was significantly associated with exam grades across the total sample. Proceeding from this, a mediation analysis revealed a significant indirect effect: Negative affect mediated the relationship between group assignment and exam grades, indicating that the OLP groups’ lower negative affect was related to better exam grades, whereas elevated negative affect in the control group corresponded with poorer exam performance. Thus, the findings point to a potential indirect pathway through which OLPs may influence academic outcomes, namely by reducing negative emotional states. These findings are an intriguing extension of prior research on OLPs and performance outcomes^25,27^ and are consistent with previous research demonstrating that elevated negative affect predicts poorer performance^81–83^. Negative affective states may impair cognitive-motivational processes critical for academic success, as they increase task-irrelevant thinking (e.g., worry), diminish attentional resources and perseverance in learning processes, and undermine motivation and academic interest^84–87^. While these underlying determinants of academic performance warrant further investigation within the OLP framework, our findings provide the first empirical evidence that OLPs may significantly influence academic functioning under stress by mitigating the impact of negative affective states.

We additionally examined whether the belief in (open-label) placebos and treatment expectations are associated with the primary outcome measures in the OLP group. Correlation analyses revealed no substantial association between belief or expectations and the effect of OLPs on psychological distress, HCC, and pre-exam negative affect, aligning with some OLP studies^25,30^ but contrasting with others^24,48^. In line with prior research^25^, belief in conventional placebo effects was not even significantly associated with belief in OLPs, suggesting that these belief constructs do not necessarily go along with each other. Nevertheless, as the underlying mechanisms that contribute to the OLP effect have not yet been entirely investigated and are critically discussed^88^, it may not be excluded that belief and expectations are relevant for OLP effects under certain conditions.

As shown in Fig. 3, the courses of the different psychological distress measures (negative affect, test anxiety, subjective stress, and exam-related stress) showed overall increasing trends in both groups during the intervention period up to the exam. As oral university exams are strong naturalistic stressors with real consequences (i.e., exam grades, possibility to fail), this increase was expected and concurs with studies reporting elevated distress during exam periods^17,89^. In contrast to previous studies focusing on pre-post OLP effects under real-life stressors^23,25,26^, our continuous assessment over the four-week intervention provides novel insights into the temporal dynamics of OLP effects. While both groups show similar levels of distress during the initial week of OLP intervention, a divergence emerges from the second week onwards. This pattern can be observed descriptively (i.e., based on a qualitative observation of the trajectories in Fig. 3), with the control group exhibiting a more pronounced increase across all four distress measures leading up to the exam day, compared to the OLP group. This consistent pattern, in line with a previous finding on OLP effects in test anxiety^27^, suggests a potential protective effect of OLPs against the accumulation of stress and adds critical knowledge on how OLP effects develop over a longer, stressful period.

Several limitations should be acknowledged. The transparent communication about a placebo intervention during exam periods does not eliminate the potential for selection bias. Highly anxious individuals may have avoided participating, thus being less represented in this sample. Furthermore, participants assigned to the control condition could have been disappointed about not receiving the OLP intervention. In the present study, 36.8% of control participants reported to be disappointed (previous OLP studies reported 27.3% and 52.5%^25,47^). While exam periods and high academic demands are typically associated with increased psychological distress^16^,^17^, this disappointment could have contributed to resentful demoralization and thus may have influenced group differences^90^. However, disappointment was not significantly associated with the primary outcome measures in the control group, suggesting that such effects are less likely to account for the observed OLP effect. Importantly, participants in the control group were offered the opportunity to receive placebo pills after completing the trial (analogous to^9^), which may have mitigated potential demoralization effects. Nevertheless, future studies may benefit from designs that further minimize potential expectation-related biases. Next, the transparent administration of placebos may contribute to response bias in self-reported data. To address this, we included multiple measures of psychological distress that showed a highly consistent pattern and were combined into a latent variable to capture shared variance and minimize measurement error. Furthermore, we observed an OLP effect on HCC as a physiological stress marker, with lower HCC values in the intervention group. Our sample size was comparatively large for an OLP study; nonetheless, replication in other study samples/cohorts would be interesting to confirm the robustness and generalizability of the findings. Future studies should incorporate objective and physiological outcome measures to further validate the effects of OLPs beyond self-reports. Moreover, while the present study highlights the regulation of negative emotional states as a pathway linking OLPs to academic performance, further research is needed to explore additional mediating mechanisms, such as changes in cognitive control, motivational dynamics, or self-efficacy beliefs under real-life stressors.

To conclude, this is the first RCT to demonstrate the beneficial effects of a four-week OLP intervention on both long-term physiological stress—measured by HCC—and on psychological distress as a latent construct within a naturalistic academic stress context. These effects persisted until the day of the oral university exam, with participants in the OLP group reporting lower negative affective states that subsequently resulted in better exam grades compared to the control group. These improvements were not associated with treatment expectations or belief in (open-label) placebos. This study provides novel empirical evidence that non-deceptive placebos can positively influence objective and physiological outcomes of stress-related measures. Despite participants’ awareness of taking inert pills, our findings highlight the potential of OLPs as an ethically justifiable and effective treatment approach that functions in the real world, with implications extending beyond self-report data.

## Data Availability

The referenced dataset that supports the findings of this study are available on the Open Science Framework (OSF), https://osf.io/9whjx.

## References

[1] Shapiro, A. K. & Shapiro, E. *The Powerful Placebo: From Ancient Priest to Modern Physician*. (Johns Hopkins University, 1997).

[2] Finniss, D. G., Kaptchuk, T. J., Miller, F. & Benedetti, F. Placebo effects: biological, clinical and ethical advances. *Lancet* **375**, 686–695 (2010). 20171404 10.1016/S0140-6736(09)61706-2 PMC2832199

[3] Darragh, M. et al. A take-home placebo treatment can reduce stress, anxiety and symptoms of depression in a non-patient population. *Aust N Z. J. Psychiatry*. **50**, 858–865 (2016). 26681262 10.1177/0004867415621390

[4] Kaptchuk, T. J., Hemond, C. C. & Miller, F. G. Placebos in chronic pain: evidence, theory, ethics, and use in clinical practic. *BMJ* m1668 (2020). 10.1136/bmj.m1668 32690477

[5] Fässler, M., Meissner, K., Schneider, A. & Linde, K. Frequency and circumstances of placebo use in clinical practice - a systematic review of empirical studies. *BMC Med.* **8**, 15 (2010). 20178561 10.1186/1741-7015-8-15 PMC2837612

[6] Blease, C. R., Bishop, F. L. & Kaptchuk, T. J. Informed consent and clinical trials: where is the placebo effect? *BMJ* **356** (j463), 1–3 (2017). 10.1136/bmj.j463 PMC6888513 28159769

[7] Lembo, A. et al. Open-label placebo vs double-blind placebo for irritable bowel syndrome: a randomized clinical trial. *Pain* **162**, 2428–2435 (2021). 33605656 10.1097/j.pain.0000000000002234 PMC8357842

[8] Schaefer, M., Harke, R. & Denke, C. Open-label placebos improve symptoms in allergic rhinitis: a randomized controlled trial. *Psychother. Psychosom.* **85**, 373–374 (2016). 27744433 10.1159/000447242

[9] Carvalho, C. et al. Open-label placebo treatment in chronic low back pain: a randomized controlled trial. *Pain* **157**, 2766–2772 (2016). 27755279 10.1097/j.pain.0000000000000700 PMC5113234

[10] Charlesworth, J. E. G. et al. Effects of placebos without deception compared with no treatment: A systematic review and meta-analysis. *J. Evid. Based Med.* **10**, 97–107 (2017). 28452193 10.1111/jebm.12251

[11] von Wernsdorff, M., Loef, M., Tuschen–Caffier, B. & Schmidt, S. Effects of open–label placebos in clinical trials: a systematic review and meta–analysis. *Sci. Rep.* **11**, 3855 (2021). 33594150 10.1038/s41598-021-83148-6 PMC7887232

[12] Spille, L., Fendel, J. C., Seuling, P. D., Göritz, A. S. & Schmidt, S. Open–label placebos - a systematic review and meta–analysis of experimental studies with non–clinical samples. *Sci. Rep.* **13**, 3640 (2023). 36871028 10.1038/s41598-023-30362-z PMC9985604

[13] Knapstad, M. et al. Trends in self-reported psychological distress among college and university students from 2010 to 2018. *Psychol. Med.* **51**, 470–478 (2021). 31779729 10.1017/S0033291719003350 PMC7958482

[14] Heumann, E., Palacio Siebe, A. V., Stock, C. & Heinrichs, K. Depressive symptoms among higher education students in germany - a systematic review and meta-analysis. *Public. Health Rev.* **45**, 1606983 (2024). 38978768 10.3389/phrs.2024.1606983 PMC11228579

[15] Matthews, G. Distress. in *Stress: Concepts, Cognition, Emotion, and Behavior*. (ed Fink, G.) 219–226 (Elsevier Academic, 2016).

[16] Pozos-Radillo, B. E., de Lourdes Preciado-Serrano, M., Acosta-Fernández, M., de los Ángeles Aguilera-Velasco, M. & Delgado-García, D. D. Academic stress as a predictor of chronic stress in university students. *Psicol. Educ.* **20**, 47–52 (2014).

[17] Losiak, W. & Losiak-Pilch, J. Cortisol awakening response, self-reported affect and exam performance in female students. *Appl. Psychophysiol. Biofeedback*. **45**, 11–16 (2020). 31486985 10.1007/s10484-019-09449-9 PMC7018672

[18] Dendle, C. et al. Medical student psychological distress and academic performance. *Med. Teach.* **40**, 1257–1263 (2018). 29355074 10.1080/0142159X.2018.1427222

[19] Schedlowski, M., Enck, P., Rief, W. & Bingel, U. Neuro-bio-behavioral mechanisms of placebo and nocebo responses: implications for clinical trials and clinical practice. *Pharmacol. Rev.* **67**, 697–730 (2015). 26126649 10.1124/pr.114.009423

[20] Balodis, I. M., Wynne-Edwards, K. E. & Olmstead, M. C. The stress-response-dampening effects of placebo. *Horm. Behav.* **59**, 465–472 (2011). 21272586 10.1016/j.yhbeh.2011.01.004

[21] El Brihi, J., Horne, R. & Faasse, K. Prescribing placebos: an experimental examination of the role of dose, expectancies, and adherence in open-label placebo effects. *Ann. Behav. Med.* **53**, 16–28 (2019). 29547962 10.1093/abm/kay011

[22] Winkler, A., Hahn, A. & Hermann, C. The impact of pharmaceutical form and simulated side effects in an open–label–placebo RCT for improving psychological distress in highly stressed students. *Sci. Rep.* **13**, 6367 (2023). 37076557 10.1038/s41598-023-32942-5 PMC10113726

[23] Kleine–Borgmann, J. et al. Effects of open-label placebos on test performance and psychological well-being in healthy medical students: a randomized controlled trial. *Sci. Rep.* **11**, 2130 (2021). 33483552 10.1038/s41598-021-81502-2 PMC7822842

[24] Schaefer, M., Hellmann-Regen, J. & Enge, S. Effects of open-label placebos on state anxiety and glucocorticoid stress responses. *Brain Sci.* **11**, 508 (2021). 33923694 10.3390/brainsci11040508 PMC8072693

[25] Schaefer, M. & Enge, S. Open-label placebos enhance test performance and reduce anxiety in learner drivers: a randomized controlled trial. *Sci. Rep.* **14**, 6684 (2024). 38509101 10.1038/s41598-024-56600-6 PMC10954622

[26] Schaefer, M. et al. Open-label placebos reduce test anxiety and improve self-management skills: A randomized-controlled trial. *Sci. Rep.* **9**, 13317 (2019). 31527670 10.1038/s41598-019-49466-6 PMC6746734

[27] Buergler, S. et al. Imaginary pills and open–label placebos can reduce test anxiety by means of placebo mechanisms. *Sci. Rep.* **13**, 2624 (2023). 36788309 10.1038/s41598-023-29624-7 PMC9926426

[28] Saravanan, C., Kingston, R. & Gin, M. Is test anxiety a problem among medical students: a cross sectional study on outcome of test anxiety among medical students? *Int. J. Psychol. Stud.* **6**, 24–31 (2014).

[29] Tsegay, L., Shumet, S., Damene, W., Gebreegziabhier, G. & Ayano, G. Prevalence and determinants of test anxiety among medical students in Addis Ababa Ethiopia. *BMC Med. Educ.* **19**, 423 (2019). 31727023 10.1186/s12909-019-1859-5 PMC6857229

[30] Guevarra, D. A., Moser, J. S., Wager, T. D. & Kross, E. Placebos without deception reduce self-report and neural measures of emotional distress. *Nat. Commun.* **11**, 3785 (2020). 32728026 10.1038/s41467-020-17654-y PMC7391658

[31] Schneider, T., Luethi, J., Mauermann, E., Bandschapp, O. & Ruppen, W. Pain response to open label placebo in induced acute pain in healthy adult males. *Anesthesiology* **132**, 571–580 (2020). 31809325 10.1097/ALN.0000000000003076

[32] Mathur, A., Jarrett, P., Broadbent, E. & Petrie, K. J. Open-label placebos for wound healing: a randomized controlled trial. *Ann. Behav. Med.* **52**, 902–908 (2018). 30212845 10.1093/abm/kax057

[33] Olliges, E. et al. Open-label placebo administration decreases pain in elderly patients with symptomatic knee osteoarthritis - a randomized controlled trial. *Front. Psychiatry*. **13**, 853497 (2022). 35599777 10.3389/fpsyt.2022.853497 PMC9122028

[34] Gao, W. et al. Quantitative analysis of steroid hormones in human hair using a column-switching LC–APCI–MS/MS assay. *J. Chromatogr. B*. **928**, 1–8 (2013). 10.1016/j.jchromb.2013.03.008 23584040

[35] Stalder, T. & Kirschbaum, C. Analysis of cortisol in hair – State of the art and future directions. *Brain Behav. Immun.* **26**, 1019–1029 (2012). 22366690 10.1016/j.bbi.2012.02.002

[36] Stalder, T. et al. Stress-related and basic determinants of hair cortisol in humans: A meta-analysis. *Psychoneuroendocrinology* **77**, 261–274 (2017). 28135674 10.1016/j.psyneuen.2016.12.017

[37] Ullmann, E. et al. Pilot study of adrenal steroid hormones in hair as an indicator of chronic mental and physical stress. *Sci. Rep.* **6**, 25842 (2016). 27174654 10.1038/srep25842 PMC4865856

[38] Lynch, R. et al. Perceived stress and hair cortisol concentration in a study of Mexican and Icelandic women. *PLOS Glob. Public. Health*. **2**, e0000571 (2022). 36962547 10.1371/journal.pgph.0000571 PMC10021558

[39] Stetler, C. A. & Guinn, V. Cumulative cortisol exposure increases during the academic term: Links to performance-related and social-evaluative stressors. *Psychoneuroendocrinology* **114**, 104584 (2020). 31982677 10.1016/j.psyneuen.2020.104584

[40] Dickerson, S. S. & Kemeny, M. E. Acute stressors and cortisol responses: a theoretical integration and synthesis of laboratory research. *Psychol. Bull.* **130**, 355–391 (2004). 15122924 10.1037/0033-2909.130.3.355

[41] Preuß, D., Schoofs, D., Schlotz, W. & Wolf, O. T. The stressed student: influence of written examinations and oral presentations on salivary cortisol concentrations in university students. *Stress* **13**, 221–229 (2010). 20235829 10.3109/10253890903277579

[42] Ciuk, D., Troy, A. & Markera, J. Measuring emotion: self-reports vs. physiological indicators. *SSRN Electron. J.* **8**, e64959 (2015).

[43] Kemeny, M. E. The psychobiology of stress. *Curr. Dir. Psychol. Sci.* **12**, 124–129 (2003).

[44] Benedetti, F. Placebo-induced improvements: how therapeutic rituals affect the patient’s brain. *J. Acupunct. Meridian Stud.* **5**, 97–103 (2012). 22682270 10.1016/j.jams.2012.03.001

[45] Meissner, K. Placebo responses on cardiovascular, gastrointestinal, and respiratory organ functions. in *Placebo. Handbook Exp. Pharmacol.* (eds Benedetti, F., Enck, P., Frisaldi, E. & Schedlowski, M.) vol. 225, 183–203 (Springer, 2014). 10.1007/978-3-662-44519-8_11 25304533

[46] Colloca, L. & Howick, J. Placebos without deception: outcomes, mechanisms, and ethics. *Int. Rev. Neurobiol.* **138**, 219–240 (2018). 29681327 10.1016/bs.irn.2018.01.005 PMC5918690

[47] Buergler, S., Sezer, D., Gaab, J. & Locher, C. The roles of expectation, comparator, administration route, and population in open–label placebo effects: a network meta–analysis. *Sci. Rep.* **13**, 11827 (2023). 37481686 10.1038/s41598-023-39123-4 PMC10363169

[48] Leibowitz, K. A., Hardebeck, E. J., Goyer, J. P. & Crum, A. J. The role of patient beliefs in open-label placebo effects. *Health Psychol.* **38**, 613–622 (2019). 31021124 10.1037/hea0000751 PMC7640758

[49] DiStefano, C., Zhu, M. & Mîndrilă, D. Understanding and using factor scores: considerations for the applied researcher. *Pract. Assess. Res. Eval*. **14**, 1–11 (2009).

[50] Llabre, M. M. & Fitzpatrick, S. L. Revisiting measurement models in psychosomatic medicine research: a latent variable approach. *Psychosom. Med.* **74**, 169–177 (2012). 22286856 10.1097/PSY.0b013e3182433a30

[51] Lee, C. H., Cook, S., Lee, J. S. & Han, B. Comparison of two meta-analysis methods: inverse-variance-weighted average and weighted sum of z-scores. *Genomics Inf.* **14**, 173–180 (2016). 10.5808/GI.2016.14.4.173 PMC5287121 28154508

[52] Kaptchuk, T. J. et al. Placebos without deception: a randomized controlled trial in irritable bowel syndrome. *PLoS One*. **5**, e15591 (2010). 21203519 10.1371/journal.pone.0015591 PMC3008733

[53] Simpson, S. H. et al. A meta-analysis of the association between adherence to drug therapy and mortality. *BMJ* **333**, 1–6 (2006). 16790458 10.1136/bmj.38875.675486.55 PMC1488752

[54] Enge, S. et al. Comparison of hair cortisol concentrations between self- and professionally-collected hair samples and the role of five-factor personality traits as potential moderators. *Psychoneuroendocrinology* **122**, 104859 (2020). 32992135 10.1016/j.psyneuen.2020.104859 PMC7462524

[55] Wennig, R. Potential problems with the interpretation of hair analysis results. *Forensic Sci. Int.* **107**, 5–12 (2000). 10689559 10.1016/s0379-0738(99)00146-2

[56] Zhu, M. et al. Simultaneous LC-MS/MS quantification of glucocorticoids, melatonin and its metabolites in hair. *J. Chromatogr. B*. **1196**, 123217 (2022). 10.1016/j.jchromb.2022.123217 35301171

[57] Breyer, B. & Bluemke, M. *Deutsche Version Der Positive and Negative Affect Schedule PANAS*. (GESIS - Leibniz-Institut für Sozialwissenschaften, 2016).

[58] Hodapp, V., Rohrmann, S. & Ringeisen, T. *Prüfungsangstfragebogen*. (Hogrefe, Göttingen, 2011).

[59] Nilges, P. & Essau, C. *DASS. Depressions-Angst-Stress-Skalen - Deutschsprachige Kurzfassung*. (ZPID, 2021).

[60] Klumbies, E., Braeuer, D., Hoyer, J. & Kirschbaum, C. The reaction to social stress in social phobia: discordance between physiological and subjective parameters. *PLoS ONE*. **9**, e105670 (2014). 25153526 10.1371/journal.pone.0105670 PMC4143269

[61] Staufenbiel, S. M., Penninx, B. W., de Rijke, Y. B. & van den Akker, E. L. van Rossum, E. F. Determinants of hair cortisol and haircortisone concentrations in adults. *Psychoneuroendocrinology* **60**, 182–194 (2015). 26176863 10.1016/j.psyneuen.2015.06.011

[62] Chida, Y. & Steptoe, A. Cortisol awakening response and psychosocial factors: a systematic review and meta-analysis. *Biol. Psychol.* **80**, 265–278 (2009). 19022335 10.1016/j.biopsycho.2008.10.004

[63] Fekedulegn, D. B. et al. Area under the curve and other summary indicators of repeated waking cortisol measurements. *Psychosom. Med.* **69**, 651–659 (2007). 17766693 10.1097/PSY.0b013e31814c405c

[64] Pruessner, J. C., Kirschbaum, C., Meinlschmid, G. & Hellhammer, D. H. Two formulas for computation of the area under the curve represent measures of total hormone concentration versus time-dependent change. *Psychoneuroendocrinology* **28**, 916–931 (2003). 12892658 10.1016/s0306-4530(02)00108-7

[65] Hu, L. T. & Bentler, P. M. Cutoff criteria for fit indexes in covariance structure analysis: Conventional criteria versus new alternatives. *Struct. Equ Model.* **6**, 1–55 (1999).

[66] Drapeau, A., Marchand, A. & Forest, C. Gender differences in the age-cohort distribution of psychological distress in Canadian adults: findings from a national longitudinal survey. *BMC Psychol.* **2**, 25 (2014).

[67] Thomsen, D. K., Mehlsen, M. Y., Viidik, A., Sommerlund, B. & Zachariae, R. Age and gender differences in negative affect—Is there a role for emotion regulation? *Personal Individ Differ.* **38**, 1935–1946 (2005).

[68] Wosu, A. C., Valdimarsdóttir, U., Shields, A. E., Williams, D. R. & Williams, M. A. Correlates of cortisol in human hair: implications for epidemiologic studies on health effects of chronic stress. *Ann. Epidemiol.* **23**, 797–811 (2013). 24184029 10.1016/j.annepidem.2013.09.006 PMC3963409

[69] Ouellet-Morin, I. et al. Validation of an adapted procedure to collect hair for cortisol determination in adolescents. *Psychoneuroendocrinology* **70**, 58–62 (2016). 27164223 10.1016/j.psyneuen.2016.05.002

[70] Frisch, N. et al. Exploring reference values for hair cortisol: hair weight versus hair protein. *Ther. Drug Monit.* **42**, 902–908 (2020). 33186335 10.1097/FTD.0000000000000779

[71] Bae, Y. J. et al. Reference intervals of nine steroid hormones over the life-span analyzed by LC-MS/MS: Effect of age, gender, puberty, and oral contraceptives. *J. Steroid Biochem. Mol. Biol.* **193**, 105409 (2019). 31201927 10.1016/j.jsbmb.2019.105409

[72] Ueland, G. Å. et al. Adrenal steroid profiling as a diagnostic tool to differentiate polycystic ovary syndrome from nonclassic congenital adrenal hyperplasia: pinpointing easy screening possibilities and normal cutoff levels using liquid chromatography tandem mass spectrometry. *Fertil. Steril.* **118**, 384–391 (2022). 35725670 10.1016/j.fertnstert.2022.05.012

[73] Okutan, S. et al. Determination of cortisol cut-off limits and steroid dynamics in the ACTH stimulation test: a comparative analysis using Roche Elecsys Cortisol II immunoassay and LC-MS/MS. *Endocrine* **85**, 321–330 (2024). 38460071 10.1007/s12020-024-03752-0 PMC11246257

[74] Eisenhofer, G. et al. Reference intervals for plasma concentrations of adrenal steroids measured by LC-MS/MS: Impact of gender, age, oral contraceptives, body mass index and blood pressure status. *Clin. Chim. Acta*. **470**, 115–124 (2017). 28479316 10.1016/j.cca.2017.05.002 PMC5504266

[75] Kline, R. B. *Principles and Practice of Structural Equation Modeling* (The Guilford Press, 2016).

[76] Davies, E. B., Richard, M. & Cris, G. Computer-delivered and web-based interventions to improve depression, anxiety, and psychological well-being of university students: A systematic review and meta-analysis. *J. Med. Internet Res.* **16**, e130 (2014). 24836465 10.2196/jmir.3142 PMC4051748

[77] Maier, L. J., Liechti, M. E., Herzig, F. & Schaub, M. P. To dope or not to dope: neuroenhancement with prescription drugs and drugs of abuse among swiss university students. *PLoS One*. **8**, e77967 (2013). 24236008 10.1371/journal.pone.0077967 PMC3827185

[78] Franke, A. G., Northoff, R. & Hildt, E. The case of pharmacological neuroenhancement: medical, judicial and ethical aspects from a german perspective. *Pharmacopsychiatry* **48**, 256–264 (2015). 26252723 10.1055/s-0035-1559640

[79] Gherardi-Donato, E. C. D. S. et al. Mindfulness practice reduces hair cortisol, anxiety and perceived stress in university workers: randomized clinical trial. *Healthcare* **11**, 2875 (2023). 37958019 10.3390/healthcare11212875 PMC10648523

[80] Iglesias, S. et al. Hair cortisol: A new tool for evaluating stress in programs of stress management. *Life Sci.* **141**, 188–192 (2015). 26454227 10.1016/j.lfs.2015.10.006

[81] Gillet, N., Vallerand, R. J., Lafrenière, M. A. K. & Bureau, J. S. The mediating role of positive and negative affect in the situational motivation-performance relationship. *Motiv Emot.* **37**, 465–479 (2013).

[82] Kaplan, S., Bradley, J. C., Luchman, J. N. & Haynes, D. On the role of positive and negative affectivity in job performance: A meta-analytic investigation. *J. Appl. Psychol.* **94**, 162–176 (2009). 19186902 10.1037/a0013115

[83] Pekrun, R., Elliot, A. J. & Maier, M. A. Achievement goals and achievement emotions: testing a model of their joint relations with academic performance. *J. Educ. Psychol.* **101**, 115–135 (2009).

[84] Assor, A., Kaplan, H., Kanat-Maymon, Y. & Roth, G. Directly controlling teacher behaviors as predictors of poor motivation and engagement in girls and boys: The role of anger and anxiety. *Learn. Instr*. **15**, 397–413 (2005).

[85] Febrilia, I., Warokka, A. & Abdullah, H. H. University students’ emotion state and academic performance: new insights of managing complex cognitive. *J. E-Learn High. Educ.* **2011**, 1–15 (2011).

[86] Pekrun, R., Goetz, T., Titz, W. & Perry, R. P. Academic emotions in students ’ self-regulated learning and achievement: a program of quantitative and qualitative research. *Educ. Psychol.* **37**, 91–105 (2002).

[87] Pekrun, R. & Stephens, E. J. Academic emotions. In *APA Educational Psychology Handbook* (eds Harris, K. R. et al.) 3–31 (American Psychological Association, 2012).

[88] Kaptchuk, T. J. Open-label placebo: reflections on a research agenda. *Perspect. Biol. Med.* **61**, 311–334 (2018). 30293971 10.1353/pbm.2018.0045

[89] O’Flynn, J., Dinan, T. G. & Kelly, J. R. Examining stress: an investigation of stress, mood and exercise in medical students. *Ir. J. Psychol. Med.* **35**, 63–68 (2018). 30115207 10.1017/ipm.2017.54

[90] Faasse, K. & Petrie, K. J. The nocebo effect: patient expectations and medication side effects. *Postgrad. Med. J.* **89**, 540–546 (2013). 23842213 10.1136/postgradmedj-2012-131730

